# An overview of alcohol screening and treatment programs in the Mexican health system

**DOI:** 10.1186/1940-0640-8-S1-A77

**Published:** 2013-09-04

**Authors:** Marcela Tiburcio-Sainz, Guillermina Natera-Rey

**Affiliations:** 1 National Institute of Psychiatry, Ramón de la Fuente Muñiz, Mexico City, Mexico

## 

As in many other countries, the cost of alcohol abuse to the Mexican health system is considerable. The main contribution to disease burden is related to morbidity and mortality associated with liver cirrhosis, accidents, and violence, including homicide. Moreover, the largest share of alcohol-related problems is caused by those who drink heavily but do not fulfill dependence criteria; consequently, this group is not properly identified and do not get treatment, since the services available are aimed at treating people with dependence. The most frequently used services are those of 12-step groups, followed by alcohol detoxification. However, in Mexico there are several initiatives to test evidence-based programs for the prevention, detection, and treatment of alcohol abuse. The most significant initiatives are reviewed in this paper highlighting its relevance in the development and implementation of public policies in Mexico, such as a solid e-learning program to train health professionals on screening and treat alcohol abuse problems; research projects to test the effectiveness of various interventions, centered primarily on the management of problem drinkers; and brief intervention for the relatives of heavy drinkers, among others. It is hoped that these initiatives will have a significant impact on the prevention, screening, and treatment of alcohol abuse, but there are tasks that remain; for instance, we need a better understanding of the barriers to implementation. Greater efforts are needed to broaden the range of alcohol screening and treatment programs, improve their efficacy and cost-effectiveness in different contexts, and encourage their adoption in the national health system.

